# Beyond Depression: The Role of Antidepressants in Managing Chronic Temporomandibular Disorders. A Systematic Review

**DOI:** 10.1111/joor.13971

**Published:** 2025-04-04

**Authors:** Takara Dei, Kennedy Galloway, Nathalia Carolina Fernandes Fagundes, Janice Y. Kung, Nathan P. Beahm, Reid Friesen

**Affiliations:** ^1^ Faculty of Pharmacy and Pharmaceutical Sciences University of Alberta Edmonton Alberta Canada; ^2^ Department of Physiology, Faculty of Medicine and Dentistry, College of Health Sciences University of Alberta Edmonton Canada; ^3^ Mike Petryk School of Dentistry, Faculty of Medicine and Dentistry, College of Health Sciences University of Alberta Edmonton Canada; ^4^ Geoffrey and Robyn Sperber Health Sciences Library University of Alberta Edmonton Canada

**Keywords:** antidepressants, orofacial pain, temporomandibular disorders

## Abstract

**Background:**

Chronic temporomandibular disorder (TMD) pain significantly impairs quality of life and lacks universally effective treatments. Antidepressants, traditionally used for mood disorders, have shown potential in managing chronic pain conditions. This systematic review evaluates the efficacy and safety of antidepressants for chronic TMD pain management.

**Methods:**

Eligibility criteria: Included randomised controlled trials (RCTs) assessing antidepressants for chronic TMD pain in adults, reporting pain reduction or functional improvement as outcomes. *Information sources*: Searches were conducted in MEDLINE, Embase, CINAHL, Scopus, Web of Science, and Cochrane Library through April 2024. *Risk of bias*: The Cochrane Risk of Bias 2 tool was used to assess study quality. *Synthesis of results*: Narrative synthesis was performed due to heterogeneity in interventions and outcomes.

**Results:**

Included studies: Seven RCTs with sample sizes ranging from 12 to 80 participants. Studies evaluated various antidepressants, including amitriptyline, duloxetine, nortriptyline, and citalopram, alone or combined with non‐pharmacological treatments. Synthesis of results: Amitriptyline and duloxetine demonstrated significant reductions in pain intensity when used in combination therapies. Functional improvements, such as increased mouth opening, were observed in some studies. Side effects, particularly with duloxetine, were more frequent than with placebo. Variability in study designs, populations, and outcome measures limited comparability. Small sample sizes, short follow‐up durations, and heterogeneity in interventions and outcomes reduced the strength of evidence.

**Conclusion:**

Antidepressants, particularly when combined with non‐pharmacological treatments, may enhance pain relief and functional outcomes for chronic TMD pain. However, high‐quality, long‐term studies are needed.

## Graphical abstract

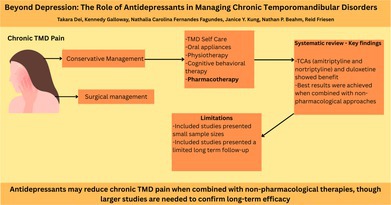

## 1 Background

Temporomandibular disorders (TMDs) represent a group of musculoskeletal conditions that affect 5%–12% of the population and are a primary cause of orofacial pain [1, 2]. TMD‐related pain is commonly classified into two primary subtypes based on the source of pain: arthrogenous and myogenous pain. Arthrogenous pain originates from the temporomandibular joint (TMJ) and is typically linked to structural abnormalities, degenerative changes, or inflammation within the joint. Patients with arthrogenous pain often present with joint tenderness, crepitus, and discomfort that worsens with jaw movement. Myogenous pain, in contrast, originates from the masticatory muscles and is typically described as diffuse, dull, and aching. It is often accompanied by muscle tenderness, fatigue, and stiffness. This type of pain frequently coexists with other chronic pain conditions and is significantly influenced by psychosocial factors such as stress and anxiety [3].

TMD pain is classified as acute or chronic. Acute pain typically arises suddenly due to trauma, overuse, or inflammation and resolves within 3 months with appropriate management. In contrast, chronic TMD pain persists beyond 3 months and is multifactorial, involving peripheral and central mechanisms, psychological factors, and functional impairments [4]. It significantly impacts quality of life, affecting physical function, psychological well‐being, and social participation.

Unlike acute pain, chronic TMD pain often extends beyond localised structural abnormalities, involving more complex pain‐processing mechanisms. Nociplastic pain, a term introduced by the International Association for the Study of Pain (IASP) in 2016, describes pain arising from altered central nervous system processing rather than tissue injury or inflammation [5]. Many cases of chronic TMD pain exhibit nociplastic characteristics, such as widespread, fluctuating pain with central sensitisation features—including fatigue, sleep disturbances, mood disorders, and multisensory hypersensitivity [5, 6]. This overlap highlights the need for management strategies that address both peripheral and central pain mechanisms.

Chronic TMD pain is typically managed using a conservative approach initially, with more invasive treatments considered based on the patient's response [4]. Conservative therapies include physiotherapy, psychotherapy, pharmacotherapy, and oral appliance therapy, all of which aim to reduce pain and improve function with varying degrees of success [7]. Oral appliances, in particular, are custom‐fitted devices worn intraorally—often during sleep—to help decrease jaw muscle activity, alleviate pain, and enhance jaw function. When conservative treatments prove insufficient, more invasive interventions such as trigger point injections, arthroscopy, or open joint surgery may be considered [7, 8]. However, no single treatment has demonstrated universal efficacy, leading to ongoing exploration of alternative pharmacological options, including antidepressants.

Given the complex and multifactorial nature of chronic TMD pain, pharmacological treatments that target central pain processing have been considered as treatment options. Antidepressants, traditionally used for mood disorders, have demonstrated efficacy in managing various chronic pain conditions [9]. Duloxetine, a serotonin‐norepinephrine reuptake inhibitor (SNRI), has been studied for its analgesic effects in fibromyalgia, diabetic neuropathy, and chronic osteoarthritis pain [10]. Likewise, tricyclic antidepressants (TCAs), including amitriptyline, have shown effectiveness in neuropathic pain and other chronic pain conditions, supporting their potential application in TMD pain treatment [11].

Since some cases of chronic TMD pain exhibit features of nociplastic pain, centrally acting agents like antidepressants may provide therapeutic benefits that extend beyond conventional treatments. By modulating neurotransmitters like serotonin and norepinephrine, these medications not only influence pain perception but may also help address associated symptoms such as mood disturbances and sleep disruptions. Given their dual impact on pain processing and psychological comorbidities, antidepressants represent a potential option for patients with chronic TMD pain who do not achieve sufficient relief with standard conservative approaches. Table 1 summarises commonly prescribed antidepressants, their indications, and their mechanisms of action.

**TABLE 1:** Common antidepressants and their associated indication and mechanism of action.

Drug	Indications	Mechanism of action
Tricyclic Antidepressants (TCAs) (e.g., amitriptyline, nortriptyline, desipramine)	FDA‐approved for the treatment of depression; also prescribed off‐label for chronic pain, insomnia, obsessive‐compulsive disorder (OCD), migraine prophylaxis, and anxiety	Increases the concentration of norepinephrine and serotonin in the brain, enhancing mood and regulating depression. This drug may also block pain signals in the spinal cord or inhibit peripheral pain signals, providing relief for chronic pain [12, 13]
Selective Serotonin Reuptake Inhibitors (SSRIs) (e.g., fluoxetine, sertraline, citalopram, escitalopram, paroxetine, fluvoxamine)	FDA‐approved for depression, OCD, bulimia nervosa, panic disorder, PTSD, social anxiety disorder, premenstrual dysphoric disorder, and generalised anxiety disorder. Off‐label uses include binge eating disorder, chronic pelvic pain, rheumatoid arthritis, and premature ejaculation	Inhibits the reuptake of serotonin in the brain, increasing serotonin levels. The precise mechanism for pain relief remains unclear [14, 15]
Serotonin‐Norepinephrine Reuptake Inhibitors (SNRIs) (e.g., venlafaxine, desvenlafaxine, duloxetine, levomilnacipran, milnacipran)	FDA‐approved for depression, anxiety, ADHD, chronic pain, diabetic peripheral neuropathic pain, bipolar disorder, fibromyalgia, and chronic musculoskeletal pain	Enhances serotonergic and noradrenergic activity in the CNS. The precise pain relief mechanism is partially understood but thought to involve amplified central pain modulation [16, 17, 18]
Norepinephrine‐Dopamine Reuptake Inhibitors (NDRIs) (e.g., bupropion)	FDA‐approved for major depressive disorder, seasonal affective disorder, and smoking cessation. Off‐label uses include ADHD, bipolar disorder, and SSRI‐induced sexual dysfunction	Treats depression by inhibiting the reuptake of norepinephrine and dopamine, enhancing their levels in the brain, and antagonising nicotinic acetylcholine receptors, which improves mood and motivation without significant serotonergic effects [19]
Monoamine Oxidase Inhibitors (MAOIs) (e.g., phenelzine, tranylcypromine, isocarboxazid, selegiline)	FDA‐approved for major depressive disorder and Parkinsonian syndrome	Blocks the monoamine oxidase enzyme, increasing levels of serotonin, norepinephrine, dopamine, and epinephrine in the CNS, thereby alleviating depressive symptoms [20, 21]
Serotonin Antagonists and Reuptake Inhibitors (SARIs) (e.g., trazodone, nefazodone)	FDA‐approved for major depressive disorder. Off‐label uses include insomnia (for sleep onset and maintenance) and dementia‐associated agitation	Inhibiting serotonin reuptake and acting as a serotonin receptor antagonist, primarily at 5‐HT2A receptors, while also having sedative effects due to its antihistaminergic and alpha‐adrenergic blocking properties [20, 22]

This systematic review aims to explore the role of antidepressants in managing TMD pain, addressing a critical gap in understanding their efficacy and clinical application for this unique pain condition.

## 2 Methods

### 2.1 Protocol and Registration

The reporting of this systematic review was guided by the standards of the Preferred Reporting Items for Systematic Review and Meta‐Analysis (PRISMA) Statement [23]. The review protocol was registered with the International Prospective Register of Systematic Reviews (PROSPERO) under the registration number CRD42024524578. The protocol can be accessed through https://www.crd.york.ac.uk/prospero/. No external funding was provided for this study.

### 2.2 Eligibility Criteria

The scope of this systematic review was defined using the PICO framework, which also guided the definition of the eligibility criteria:

- Population (P): Adults diagnosed with chronic TMD pain, including individuals with myofascial pain, arthralgia, or other TMD‐related chronic pain conditions.
- Intervention (I): The use of antidepressants. Studies where antidepressants were combined with non‐pharmacological treatments were also included.
- Comparator (C): Any treatment that does not include antidepressants, including placebo, non‐systemic pharmacological treatments (e.g., NSAIDs, muscle relaxants), non‐pharmacological interventions (e.g., oral appliances, trigger point injections, physiotherapy, arthrocentesis with or without additional medications such as corticosteroids), or standard care without antidepressants.
- Outcome (O): Primary outcomes included pain reduction (e.g., measured by VAS, NRS) and functional improvement (e.g., mouth opening, quality of life). Secondary outcomes included adverse effects and improvement in associated symptoms, such as depression or anxiety.

Studies were included if they examined the use of antidepressants for managing chronic TMD pain, enrolled adult participants (≥ 18 years) diagnosed with chronic TMD—defined as pain persisting for at least 3 months—based on the Diagnostic Criteria for Temporomandibular Disorders (DC/TMD) with a diagnosis of myalgia or arthralgia, and utilised a randomised controlled trial (RCT) design; reported primary outcomes related to pain reduction, quality of life, or functional improvement; published in peer‐reviewed journals and available in the English language.

Studies were excluded if they met the following criteria: non‐randomised studies, observational studies, case reports, reviews, and editorials; studies focused on other types of orofacial pain (non‐TMD); studies involving participants with severe psychiatric disorders unrelated to TMD.

### 2.3 Search Strategy

A comprehensive search strategy was developed and executed in collaboration with the medical librarian (JYK) to identify relevant studies. On 22 April 2024, searches were conducted across multiple electronic databases, including Ovid MEDLINE, Ovid Embase, CINAHL, Scopus, Web of Science Core Collection, and the Cochrane Library (via Wiley). Relevant keywords and controlled vocabulary were carefully selected to capture all literature about the use of antidepressants in managing chronic TMD. The search was limited to studies published in English. Full search strategies are detailed in the appendices (Table S1).

### 2.4 Study Selection

Covidence (www.covidence.org), a web‐based tool for managing systematic reviews, was used to screen and select the studies. The study selection process involved two independent reviewers who screened titles and abstracts of all the unique records after duplicate removal to assess their eligibility based on the predefined inclusion and exclusion criteria. Full‐text articles were then reviewed in detail to confirm their eligibility. Discrepancies between reviewers (KG and TD) were resolved through discussion or by consulting a third reviewer (RF). Additionally, the bibliographies of included studies were reviewed for any relevant references.

### 2.5 Data Collection Process

Data extraction was carried out independently by two reviewers using a standardised form. Extracted data included bibliographic information (e.g., author, year of publication, country), participant demographics (e.g., sample size, age, diagnosis), intervention details (e.g., type of antidepressant, dosage, duration), comparator interventions, and reported outcomes (e.g., pain reduction, quality of life, adverse effects). Any discrepancies in data extraction were resolved through consensus or consultation with a third reviewer. In case of missing data, the corresponding author of the included study was contacted by email for up to three consecutive weeks, in which one contact attempt per week was performed.

In addition to outcomes, data were extracted on the following variables: participant characteristics (age, gender, diagnostic criteria, and duration of TMD pain), intervention details (type, dosage, and duration of antidepressant treatment), comparator details (placebo or non‐pharmacological treatments), and study characteristics (design, sample size, and setting). Efforts were made to contact study authors to clarify missing or ambiguous data, though this was not always successful.

### 2.6 Risk of Bias Assessment and Applicability

The risk of bias in the included studies was assessed using the Cochrane Risk of Bias 2 (RoB 2) tool, which evaluates key domains such as random sequence generation, allocation concealment, blinding of participants and outcome assessors, completeness of outcome data, and selective reporting. Based on these criteria, each study was categorised as low risk of bias, some concerns, or a high risk of bias [24]. Additionally, the applicability of the study findings was assessed to determine the generalisability of the populations, interventions, and outcomes to clinical practice.

### 2.7 Summary Measures and Data Synthesis

The data collected was summarised in Table 2, and a narrative synthesis was prepared. The feasibility of a quantitative synthesis (i.e., meta‐analysis) was assessed for the included studies. However, a meta‐analysis could not be performed due to significant clinical heterogeneity across included studies (i.e., use of different antidepressants between studies) and missing data (i.e., some studies reported their findings in a graphical presentation only). In case of missing data, attempts have been made to contact the corresponding authors, but they were unsuccessful. Clinical heterogeneity across studies, including variations in antidepressant classes, comparator interventions, and outcome measures, was identified during data synthesis. While formal methods such as subgroup analysis or meta‐regression were not performed due to the limited number of comparable studies, heterogeneity was narratively explored by grouping studies according to antidepressant class and outcome domains. Differences in study design, population characteristics, and intervention protocols were also qualitatively examined to identify potential sources of variability in results.

**TABLE 2:** Summary of descriptive characteristics of included articles.

Author/year/country/journal	Population studied, sample size, *N*, age range or mean of age	Intervention characteristics				Outcomes in intervention group(s), adverse effects, follow‐up
		Diagnosis	Intervention	Comparator	Outcome measures	
Rizzatti‐Barbosa et al. 2003 [25] Brazil/J Applied Oral Sci	Population: Female patients with signs and symptoms of chronic TMD. Excluded were pregnant/breastfeeding individuals, those with systemic diseases, and individuals on other medications at the time of the study. sample size:12 age (range/mean): ≥ 18 years of age	Chronic TMD—not specifically calibrated	25 mg amitriptyline once daily for 14 days	Placebo pill identical in size and appearance to amitriptyline 25 mg	VAS for pain and discomfort	Pain reduction: VAS baseline: 44%; post‐treatment: 10.14% (*p* = 0.01). discomfort reduction: baseline: 45.42%; post‐treatment: 12.33%. adverse effects: none reported. follow‐up: 14 days
Alajbeg et al. 2018 [26] Croatia/Acta Stomatologica Croatica	Population: Patients with chronic TMD. Excluded: systemic disease, pregnancy, cardiac issues. Sample size: 13: Group A: 4, Group B: 4, Group C: 5. Age (range or mean): Group A: 57.2 ± 8.1 years; Group B: 46.5 ± 18.1 years; Group C: 42.8 ± 12.4 years	RDC/TMD (Groups I and II)	Group A: Amitriptyline 25 mg daily for 12 weeks. Group C: Oral appliance is worn during sleep	Group B: Placebo pill identical in size and appearance to amitriptyline 25 mg	VAS for pain, OHIP‐14 for oral health‐related quality of life, and MCO for maximum comfortable opening	Pain reduction: Significant reductions in VAS in the amitriptyline group (*F* = 11.326, *p* = 0.002) and oral appliance (*F* = 7.343, *p* = 0.005). Quality of Life (OHIP‐14): Significant improvement in the amitriptyline group only (*F* = 4.417, *p* = 0.036). Comfortable mouth opening: Significant improvement in the oral appliance group compared to amitriptyline and placebo (*p* > 0.05). Adverse effects: None reported. Follow‐Up: 1st, 6th, and 12th week
Singh et al. 2018 [10] India/J Maxillofac Oral Surg	Population: Patients with TMJ pain confirmed by RDC/TMD criteria, pain duration ≥ 3 months. Exclusions: systemic diseases, pregnancy, breastfeeding, allergies. Sample size: 20 (Group A: 10, Group B: 10). Age (range or mean): ≥ 18 years	TMJ pain confirmed by RDC/TMD criteria, with emphasis on pain of non‐odontogenic origin and presence of symptoms for at least 3 months	Group B: TMJ arthrocentesis + duloxetine 30 mg BID for 12 weeks	Group A: TMJ arthrocentesis alone	BPI pain, mouth opening, painful/pain‐free jaw movement, IL‐6 levels	Pain reduction: Group B had significantly lower pain at weeks 4, 6, and 12 (*p* < 0.05). Mouth opening: Group B showed greater improvement (*p* < 0.05). IL‐6 levels: Significant reduction in both groups, no difference between groups. Adverse effects: None of the group B individuals reported any adverse effects. Follow‐up: Baseline, days 1, 5, 7, weeks 4, 6, and 12
Vinuthna et al. [27] 2020/United Kingdom/Eur J Molecular and Clin Med	Population: Patients with TMJ pain diagnosed using RDC/TMD criteria, with pain duration ≥ 3 months. Sample size: 40 (Group I: 20, Group II: 20) Age (range or mean): ≥ 18 years of age	TMJ confirmed by RDC/TMD	Group II: TMJ arthrocentesis + duloxetine 30 mg BID for 12 weeks	Group I: Arthrocentesis alone	VAS for pain, mouth opening (mm)	Pain reduction: Group II had significantly lower pain (VAS) at weeks 6 and 12 (*p* < 0.05). Mouth opening: Group II showed significantly greater improvement in maximum mouth opening at weeks 6 and 12 (*p* < 0.05). Adverse effects: None reported. Follow‐up: Days 1, 5, 7, weeks 4, 6 and 12
Tak et al. [28] 2021/India/J Indian Acad Oral Med and Radiol	Population: Patients aged 18–45 with TMD‐related myalgia or myofascial pain lasting ≥ 3 months, excluding systemic diseases, pregnancy, and medication intolerance Sample size: 40 (Group I: 20, Group II: 20) Age (range or mean): Group I: 19–44 years; Group II: 18–45 years	TMD confirmed by RDC/TMD criteria	Group II: Gabapentin 100 mg BID + Nortriptyline 10 mg for 3 weeks	Group I: Gabapentin alone 100 mg BID for 3 weeks	VAS for pain, maximum interincisal mouth opening, and muscle tenderness sites	Pain reduction: VAS scores decreased significantly more in Group II (*p* < 0.001 at week 3). Mouth opening: Group II showed significantly greater improvement by week 3 (41.5 mm vs. 38.35 mm; *p* = 0.003). Muscle tenderness: Greater reduction in tender sites in Group II (*p* = 0.008) Adverse effects: None reported
Ferreira et al. [29] 2023/Brazil/J Oral Rehabil	Population: Patients aged ≥ 18 with painful TMD (arthralgia, myalgia, or TMD headache) for ≥ 3 months, excluding uncontrolled systemic disorders, medication allergies, pregnancy, and recent TMD treatment. Sample size: 80 (SM‐duloxetine: 40, SM‐placebo: 40) Age (range or mean): SM‐duloxetine: 38.8 ± 10.6 years; SM‐placebo: 39.7 ± 11.2 years	TMD confirmed by DC/TMD	SM‐duloxetine 60 mg/day (titrated over 2–3 weeks)	Self‐management (education, self‐exercise, thermal modalities, dietary suggestions, parafunctional behaviour awareness)	Pain intensity (NRS), CPM, physical functioning, emotional factors, and safety	Pain reduction: Pain intensity decreased in both groups but no significant difference between groups (*p* = 0.82). CPM: More efficient CPM is associated with greater pain reduction, regardless of the treatment group (*p* = 0.035). Adverse effects: Higher in the SM‐duloxetine group (90% vs. 65%, *p* = 0.014). Follow‐up: 12 weeks
Sousa et al. [30] 2024/Portugal/Medicina Clínica	Population: Adults with chronic orofacial pain lasting > 6 months caused by TMD. Excluded: pregnancy, systemic diseases, adverse reactions to study drugs. Sample Size: 64 (Control: 22, Citalopram: 21, Amitriptyline: 21) Age (Range or Mean): Control: 36 years; Citalopram: 40 years; Amitriptyline: 39 years	Chronic orofacial pain caused by TMD. Specific criteria not specified	Group 2: 10 mg/day of citalopram. Group 3: 25 mg/day of amitriptyline. Both for 9 weeks	Group 1: Michigan‐style oral appliance only	VAS for pain intensity	Pain reduction: Amitriptyline group showed the greatest reduction in VAS scores: 3.3 ± 1.5 at 3 weeks, 1.5 ± 1.4 at 6 weeks, 0.9 ± 1.3 at 9 weeks (*p* = 0.007 compared to control). Citalopram group showed improvement but was not significantly different from the control group (*p* = 0.058). No significant difference between amitriptyline and citalopram (*p* = 0.692). Control group (night splint) VAS scores: 5.9 ± 1.3 at baseline, 3.9 ± 2.1 at 3 weeks, 3.4 ± 2.2 at 6 weeks, 3.1 ± 2.3 at 9 weeks. Adverse effects: Amitriptyline group: Mild adverse effects reported (dry mouth), not statistically significant. Follow‐up: 9 weeks

Abbreviations: BID, twice daily; BPI, brief pain inventory; CPM, conditioned pain modulation; NRS, numeric rating scale; SM, self‐management; VAS, visual analogue scale.

For the synthesis of results, specific effect measures were chosen based on the type of outcome being analysed. Continuous outcomes, including pain reduction and functional improvement, were analysed using mean differences (MD) or standardised mean differences (SMD), each accompanied by 95% confidence intervals (CIs). When studies presented results in varying formats, such as visual analogue score (VAS) scores reported on different scales (e.g., 10‐point or 100‐point scales), these were converted to a standardised 0–10 scale to ensure comparability across studies.

To prepare the data for synthesis, measures were standardised as necessary, and when key summary statistics, such as standard deviations, were not reported, they were imputed using available data or derived from related metrics through established statistical formulas. Efforts to contact study authors to obtain missing data were made but were often unsuccessful.

### 2.8 Level of Evidence

The overall certainty of the evidence was assessed using the GRADEpro criteria (Grading of Recommendations Assessment, Development, and Evaluation‐GRADEpro Guideline Development Tool [Software]. McMaster University and Evidence Prime, 2024). This tool assesses the level of evidence among five outcomes of this systematic review: changes in pain intensity among adults of TMD while using the following antidepressants: nortriptyline, duloxetine, amitriptyline, and citalopram. Studies were evaluated according to their study design, risk of bias, inconsistent results, indirect evidence, and imprecision [31].

## 3 Results

### 3.1 Study Selection

**FIGURE 1:**
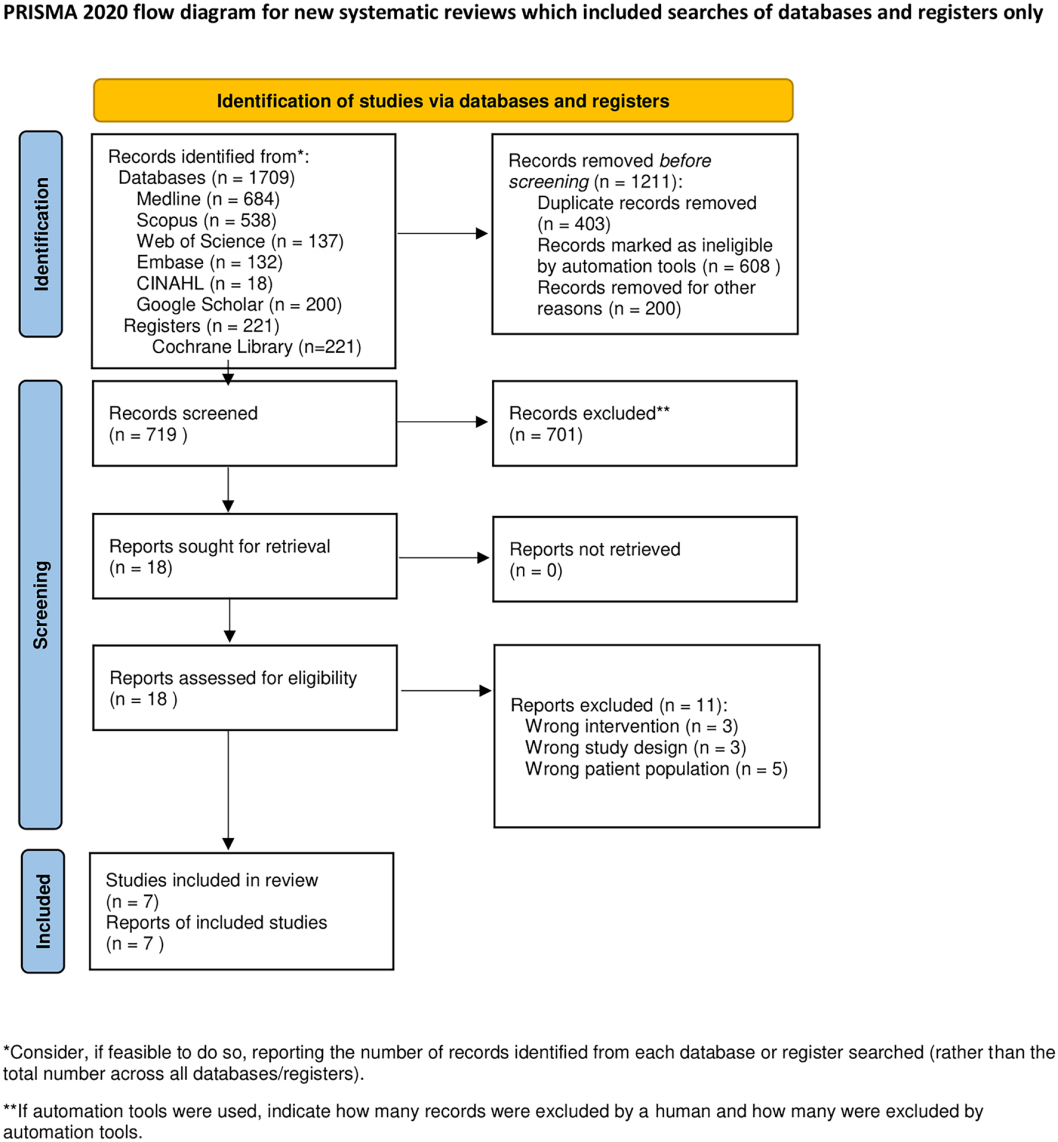
PRISMA TMD and antidepressants flowchart.

A total of 1930 records were identified through database and register searches, including Medline (684), Scopus (538), Web of Science (137), Embase (132) and CINAHL [28], Google Scholar (200), and Cochrane Library (221). The research team also conducted searches of trial registries (*n* = 221) and screened the first 200 results from Google Scholar, which has been shown to significantly overlap with Web of Science [32]. After the removal of 1211 duplicate records, 719 records were screened. Following title and abstract screening, 701 records were excluded. Full‐text retrieval was sought for 18 studies. Among the retrieved studies, 11 were excluded for the following reasons: wrong intervention (*n* = 3), wrong study design (*n* = 3), and wrong patient population due to incorrect age (*n* = 5) (Appendices, Table S2). Ultimately, seven studies met the inclusion criteria and were included in the systematic review. The flow of studies through the selection process is illustrated in the PRISMA diagram (Figure 1), which provides a detailed account of the number of records identified, screened, assessed for eligibility, and included in the review.

### 3.2 Study Characteristics

The studies included in this review encompassed a range of methodologies, patient populations, and interventions aimed at evaluating the role of antidepressants in managing chronic TMD pain. The studies were conducted across various countries, including India, Brazil, and the United Kingdom, and involved different types of antidepressants, such as duloxetine and amitriptyline. The sample sizes varied, with some studies involving small cohorts of around 12 participants, while others included up to 80 participants. The study durations and specific outcome measures differed, but all focused on assessing the impact of antidepressants on pain relief and overall patient quality of life in individuals with chronic TMD.

### 3.3 Risk of Bias Assessment

**FIGURE 2:**
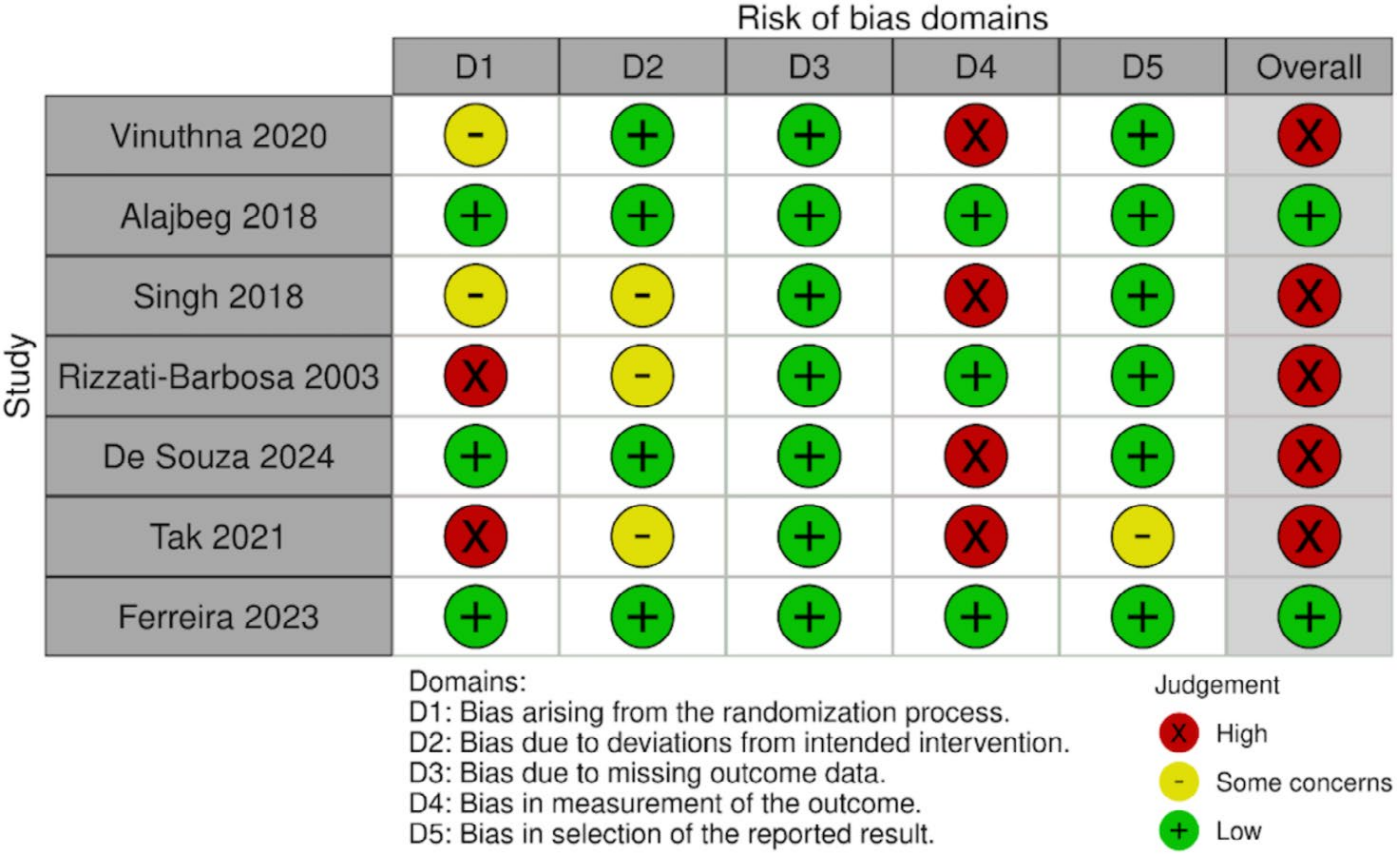
RoB assessments for each individual study included in the systematic review were evaluated across all 5 domains of the Cochrane RoB 2 tool, including overall RoB rating.

Five studies were categorised as presenting a high risk of bias, whereas the other two studies were categorised as presenting a low risk of bias. Overall, the identified risk of bias arose from potential bias in the randomisation process and in the measurement of the outcome. Also, some concerns regarding bias due to deviations from intended interventions and selection of the reported results were identified. Figure 2 summarises the assessed risk of Bias across the included studies. The certainty of evidence can be found in Table 3.

**TABLE 3:** Certainty of evidence assessment.

Certainty assessment							Summary of findings		
Participants (studies) follow‐up	Risk of bias	Inconsistency	Indirectness	Imprecision	Publication bias	Overall certainty of evidence	Study event rates (%)		Impact
							With placebo	With antidepressants	
Nortriptyline (follow‐up: 3 weeks)									
40 (1 RCT)	Very serious^c^	Not serious	Not serious	Not serious	None	⨁⨁◯◯ Low^c^	The use of Nortriptyline 10 mg, combined to Gabapentin 100 mg twice daily, might reduce pain and improve comfortable mouth opening in adults when compared to the use of Gabapentin 100 mg twice daily. However, due to the low certainty of evidence, the true effect might be markedly different		
Duloxetine (follow‐up: 12 weeks)									
114 (3 RCTs)	Serious^a^	Not serious	Not serious	Serious^d^	None	⨁⨁◯◯ Low^a^, ^d^	Contrasting results were observed across studies. The use of 60 mg of duloxetine has not shown significant changes in pain intensity when compared to a placebo in one study. In the other two studies, 30 mg of duloxetine (twice daily) when combined with arthrocentesis showed a reduction in pain intensity when compared to arthrocentesis only. Also, due to the low certainty of evidence, the true effect might be markedly different. It is not possible to make a recommendation regarding the clinical effectiveness of duloxetine for pain management among adults with TMD pain		
Amitriptyline (follow‐up: range 2 weeks to 12 weeks)									
61 (3 RCTs)	Very serious^c^	Not serious	Not serious	Serious^b^	None	⨁◯◯◯ Very low^b^, ^c^	The use of Amitriptyline 25 mg/day showed a significant reduction in pain and discomfort among adults with TMD. However, due to the low certainty of evidence, the true effect is probably markedly different		
Citalopram (follow‐up: 9 weeks)									
42 (1 RCT)	Serious^a^	Not serious	Not serious	Serious^b^	None	⨁⨁◯◯ Low^a^, ^b^	The use of citalopram 10 mg has not shown significant changes in pain intensity when compared to a control group that used a Michigan‐type nighttime occlusal oral appliance. However, due to the identified low certainty of evidence, the true effect might be markedly different		

Abbreviation: CI, confidence interval.

^a^ High risk of bias in measurement of the outcome.

^b^ A substantial variation in 95% CI (> 20%) was detected between intervention groups.

^c^ High risk of bias arising from the randomisation process and in the measurement of the outcome.

^d^ A substantial variation in 95% CI (> 20%) was observed between treatment groups in two studies.

### 3.4 Synthesis of Results

The findings from the included studies suggest that antidepressants may play a beneficial role in managing chronic TMD pain, mainly when used in combination with other therapeutic interventions. Several studies demonstrated that integrating antidepressants with non‐pharmacological approaches, such as trigger point injections or arthrocentesis, enhanced pain relief and improved functional outcomes.

For instance, duloxetine, a SNRI, enhanced pain relief and functional outcomes when added to arthrocentesis, compared to arthrocentesis alone. These findings highlight the potential for synergistic effects between pharmacological and non‐pharmacological strategies [10].

While combination therapies generally yielded better results, the effectiveness of antidepressants as standalone treatments was more variable. Amitriptyline consistently showed significant reductions in pain intensity and improved quality of life, particularly when combined with oral appliances [26, 30]. However, the evidence for citalopram was weaker, with studies reporting no significant differences in pain outcomes compared to control treatments, such as oral appliances [30].

The combination of nortriptyline with gabapentin also showed promising results. Tak et al. [28] reported that the combination significantly reduced VAS scores compared to gabapentin alone (*p* < 0.001), alongside improved mouth opening and reduced muscle tenderness (*p* < 0.05). These findings highlight the potential for antidepressants to enhance outcomes when integrated into multimodal treatment plans.

Despite these promising results, antidepressants are not without limitations. Side effects, such as those associated with duloxetine (GI disturbances, elevated blood pressure, and drowsiness), were more prevalent in some studies, with higher rates of adverse events reported when compared to placebo or standalone therapies [29]. These findings emphasise the importance of tailoring treatment plans to individual patient needs, weighing potential benefits against the risk of adverse effects.

The variability in outcomes across studies may also be influenced by differences in study design, populations, and intervention protocols. While some studies reported significant benefits, others highlighted limitations, such as small sample sizes, short follow‐up periods, and inconsistencies in diagnostic criteria.

These findings are summarised in Table 2, which provides an overview of the included studies, detailing interventions, outcomes, and key results. Overall, the evidence suggests that antidepressants, particularly when used as part of combination therapy, can improve pain management and quality of life for patients with chronic TMD pain. However, further high‐quality research is needed to establish their standalone efficacy and to refine treatment protocols.

### 3.5 Heterogeneity Among Studies

Significant heterogeneity was observed across studies, primarily due to variations in antidepressant types, intervention protocols (e.g., standalone vs. combination treatments), outcome measures (e.g., VAS, mouth opening), and follow‐up durations (ranging from 2 to 12 weeks). These differences limited the ability to compare results directly or perform a quantitative synthesis.

### 3.6 Certainty of Evidence

The certainty of the evidence regarding the effect of antidepressants on pain intensity among adults with TMD was graded as very low to amitriptyline 25 mg and low to nortriptyline 10 mg, duloxetine 60 mg, and citalopram 10 mg. These results were mainly influenced by the high risk of bias and serious imprecision identified in some studies (Table 2).

## 4 Discussion

This systematic review evaluated the efficacy of antidepressants in managing chronic TMD pain. To the best of our knowledge, this is the first systematic review to evaluate the efficacy of antidepressants in the management of chronic TMD pain. Antidepressants, such as amitriptyline, duloxetine, and nortriptyline, demonstrated some effectiveness for pain reduction, particularly when used in combination with other therapies like trigger point injections, oral appliances, or arthrocentesis. For example, duloxetine paired with arthrocentesis consistently yielded superior pain relief and functional improvements compared to arthrocentesis alone. Amitriptyline and nortriptyline also showed promising results in improving both pain and functional outcomes across multiple studies. However, the effectiveness of antidepressants as standalone treatments was less consistent, with citalopram showing limited efficacy, which is consistent with other studies showing limited efficacy for selective serotonin reuptake inhibitors (SSRIs) for chronic pain [33, 34]. These findings highlight the potential role of antidepressants as an adjunctive treatment for chronic TMD pain.

The findings of this review align with previous evidence supporting the use of antidepressants for chronic pain management beyond their traditional role in treating mood disorders. For example, Birkinshaw et al. [33] demonstrated that duloxetine effectively reduced chronic pain across various conditions, consistent with this review's findings of duloxetine providing modest pain relief in TMD‐related pain. Similarly, McQuay et al. [35] highlighted the efficacy of low‐dose amitriptyline (25 mg) in significantly reducing chronic nonmalignant pain, aligning with this review's results indicating amitriptyline's role in TMD pain relief. Combination treatments, such as pairing antidepressants with arthrocentesis or oral appliances, generally led to better outcomes than antidepressants alone. Most of the studies reviewed incorporated both pharmacological and non‐pharmacological approaches, reinforcing the reality that TMD is not solely a physical condition. These findings align with the biopsychosocial model of TMD management, highlighting the importance of addressing psychological and behavioural factors alongside physical treatments to achieve meaningful improvements.

Other literature further supports these conclusions. Ferreira et al. [36] found that SNRIs and TCAs had modest effects on chronic pain, including back pain and osteoarthritis, though these effects were not always clinically meaningful, echoing this review's findings on variable efficacy across studies. Kuijpers et al. [37] similarly reported limited evidence for antidepressants in managing chronic low back pain, citing no significant differences between antidepressants and placebo in many cases, paralleling some inconsistencies noted in TMD pain management. Ferreira et al. [36] emphasised a significant incidence of adverse effects with SNRIs, such as duloxetine, which corresponds with this review's observation of a relatively higher rate of adverse effects in the duloxetine‐treated groups.

Additionally, Goyal et al. [38] suggested that combination therapies involving antidepressants enhance pain relief, though their non‐RCT design limits reliability. This aligns with findings in this review where combination approaches, such as duloxetine with arthrocentesis or nortriptyline with gabapentin, showed better outcomes than standalone antidepressant use. The consistent efficacy of antidepressants like duloxetine and amitriptyline in conditions such as fibromyalgia and diabetic neuropathy further reinforces their observed benefits in TMD‐related pain within this review.

This alignment highlights the broader applicability of antidepressants in chronic pain management while underscoring the need for high‐quality, long‐term research to address the observed heterogeneity and methodological limitations.

### 4.1 Clinical Implications

The findings of this review underscore the potential for antidepressants to expand the therapeutic toolkit for managing chronic TMD pain. These medications offer an evidence‐based, non‐invasive treatment option for patients who are either not candidates for surgical interventions or prefer less invasive approaches. Additionally, pairing antidepressants with other therapies may maximise treatment outcomes. Clinicians should consider integrating these options into multimodal treatment plans while discussing the risks and benefits with patients.

This review highlights the importance of tailoring treatment approaches to individual patient needs, as certain antidepressants demonstrated higher incidences of adverse effects. For instance, duloxetine was associated with more frequent side effects compared to placebo, including nausea, dry mouth, fatigue, insomnia, and increased sweating, emphasising the need for careful patient selection and monitoring. The results advocate for the inclusion of antidepressants as adjunctive therapies, potentially improving the quality of life for TMD patients.

### 4.2 Strengths and Limitations

This systematic review has several notable strengths. First, it adhered to PRISMA guidelines, ensuring a transparent and methodologically robust process for study selection, data extraction, and synthesis. The inclusion of a diverse range of antidepressants, study designs, and therapeutic approaches provides a comprehensive overview of the potential role of antidepressants in managing chronic TMD pain. Additionally, the use of the Cochrane Risk of Bias 2 tool enabled a rigorous assessment of methodological quality, enhancing the reliability of the findings.

However, there are important limitations to consider. The review included only seven high‐quality RCTs, all of which had fairly small sample sizes, with the largest including 80 patients. Five of these studies exhibited a high risk of bias. One study had a particularly small sample size of only 12 participants, which limits the generalisability and statistical power of its findings. Furthermore, approximately 20 potentially relevant studies were excluded due to lack of access, introducing the potential for selection bias. The studies included in the review also demonstrated considerable heterogeneity in terms of interventions, outcome measures, and follow‐up durations. For example, not all studies used standardised pain scales, such as the VAS, which precluded a meta‐analysis. Other factors that prevented conducting a meta‐analysis include variations in the interventions used across the studies and inconsistencies in how the data was reported. Most studies also had short intervention and follow‐up durations, failing to account for the long‐term efficacy and safety of antidepressant use in this population. Due to the variability among the included studies, we were unable to assess the effectiveness of antidepressants separately for patients with myogenous versus arthrogenous TMD. Recognising this distinction is essential for future research and could help refine treatment approaches.

A further limitation relates to the review process itself. The search strategy excluded grey literature and non‐English language studies, which may have increased the risk of publication bias. While two reviewers independently screened studies, subjective bias in study selection and data extraction cannot be entirely ruled out. Additionally, data extraction was hindered by incomplete reporting in some studies, and efforts to contact authors for clarification were often unsuccessful. The inability to conduct a meta‐analysis due to heterogeneity restricted the ability to provide pooled effect estimates, and while a narrative approach was used to assess the certainty of evidence, formal frameworks were not consistently applied.

Finally, several included studies were assessed as having a high risk of bias due to issues such as inadequate randomisation, lack of blinding, or incomplete outcome data. These methodological shortcomings reduce the certainty of the evidence and highlight the need for more rigorous study designs in future research. Despite these limitations, the review provides valuable insights into the potential role of antidepressants in chronic TMD pain management and underscores the need for high‐quality, standardised studies to better inform clinical practice.

### 4.3 Future Directions

Future research should address the gaps identified in this review to clarify the role of antidepressants in chronic TMD pain management. Comparative studies evaluating different antidepressant classes, optimal dosing, treatment duration, and long‐term safety are needed. Standardised outcome measures, such as VAS or NRS, should be adopted to facilitate comparisons and meta‐analyses. Investigating patient‐centered outcomes, genetic factors, and predictors of response may support more personalised treatment approaches.

Further research should also focus on determining the optimal timing for introducing antidepressants in TMD management. Understanding when these medications provide the most benefit—whether as an early intervention in patients with central sensitisation features or as a later option for those unresponsive to conservative care—could help refine treatment protocols. Establishing clear clinical guidelines for incorporating antidepressants may not only improve pain and function but also reduce the need for more invasive treatments by effectively addressing central pain mechanisms before symptoms become refractory.

## 5 Conclusions

This systematic review suggests that antidepressants may reduce pain and improve function in chronic TMD patients, particularly when combined with other treatments. Medications such as TCAs and duloxetine showed potential benefits. However, the evidence remains limited by small sample sizes, short follow‐up periods, and study heterogeneity. Future research should refine dosing, evaluate long‐term safety, focus on patient‐centered outcomes, and assess the role of comorbidities to guide clinical practice. Until more robust evidence is available, antidepressants should be integrated into a personalised, multimodal treatment approach rather than used as a standalone therapy.

## Author Contributions

**Takara Dei:** conceptualisation, methodology, data curation, formal analysis, writing – original draft, writing – review and editing. **Kennedy Galloway:** data curation, screening and study selection, writing – original draft, writing – review an editing. **Nathalia Carolina Fernandes Fagundes:** formal analysis, writing – review and editing. **Janice Y. Kung:** search strategy development, literature search execution, data curation, writing – review and editing. **Nathan P. Beahm:** writing – original draft, writing – review and editing. **Reid Friesen:** conceptualisation, supervision, methodology, data analysis, writing – original draft, writing – review and editing, project administration. All authors reviewed and approved the final manuscript and agree to be accountable for all aspects of the work, ensuring its accuracy and integrity.

## Conflicts of Interest

The authors declare no conflicts of interest.

### Peer Review

The peer review history for this article is available at https://www.webofscience.com/api/gateway/wos/peer‐review/10.1111/joor.13971.

## Supporting information


Data S1.

## Data Availability

The following materials used in this systematic review are available upon request: template data collection forms, extracted data from included studies, data used for analyses, and analytic code. These materials are not publicly posted but can be obtained by contacting the corresponding author, Dr. Reid Friesen, at rtfriese@ualberta.ca.
