# Supplementary material for: Beyond Depression: The Role of Antidepressants in Managing Chronic Temporomandibular Disorders. A Systematic Review
Source: J Oral Rehabil. 2025 Apr 4;52(6):923–36. doi: 10.1111/joor.13971 (PMC12102682; doi:10.1111/joor.13971)
Supplement: Supplementary file 1 — Data S1. [file JOOR-52-923-s001.docx]

**APPENDICES**

Table S1: Search Strategies adopted to each database.

| **Database** | **Search Strategy** |
| --- | --- |
| MEDLINE  Ovid MEDLINE(R) ALL 1946 to April 19, 2024 | 1. exp ANTIDEPRESSIVE AGENTS/  2. exp MONOAMINE OXIDASE INHIBITORS/  3. exp NEUROTRANSMITTER UPTAKE INHIBITORS/  4. ((serotonin or norepinephrine or noradrenaline or neurotransmitter* or dopamin*) and (uptake or reuptake or re uptake)).tw.  5. (noradrenerg* or antiadrenergic or anti adrenergic or SSRI* or SNRI* or NARI* or SARI* or NDRI* or TCA* or tricyclic* or tetracyclic* or heterocyclic or pharmacotherap* or psychotropic).tw.  6. (antidepress* or anti-depress*).tw.  7. (MAOI* or RIMA).tw.  8. monoamine oxidase inhibit*.tw.  9. (Agomelatine or Amoxapine or Amineptine or Amitriptylin* or Amitriptylinoxide or Atomoxetine or Befloxatone or Benactyzine or Brofaromin*).tw.  10. (Bupropion or Amfebutamone or Butriptylin* or Caroxazone or Cianopramin* or Cilobamin* or Cimoxatone or Citalopram or Chlorimipramin* or Clomipramin* or Chlomipramin* or Clomipramine).tw.  11. (Clorgyline or Clovoxamin* or "CX157" or Tyrima or Tririma or Demexiptilin* or Deprenyl or Desipramin* or Pertofrane or Desvenlafaxine or Dibenzepin or Diclofensin* or Dimetacrin* or Dosulepin or Dothiepin or Doxepin or Duloxetine or Desvenlafaxine or "DVS-233" or Escitalopram or Etoperidone or Femoxetin* or Fluotracen or Fluoxetine or Fluvoxamin*).tw.  12. (Hyperforin or Hypericum or St John* or Imipramin* or Iprindole or Iproniazid* or Ipsapirone or Isocarboxazid* or Levomilnacipran or Lofepramin* or "Lu AA21004" or Vortioxetine or "Lu AA24530" or Tedatioxetine or "LY2216684" or Edivoxetine or Maprotilin* or Medifoxamin* or Melitracen or Metapramin* or Mianserin or Milnacipran or Minaprin* or Mirtazapin* or Moclobemide).tw.  13. (Nefazodone or Nialamide or Nitroxazepine or Nomifensin* or Norfenfluramin* or Nortriptylin* or Noxiptilin* or Opipramol or Paroxetine or Phenelzine or Pheniprazine or Pipofezine or Pirlindole or Pivagabine or Pizotyline or Propizepine or Protriptylin* or Quinupramine or Reboxetine or Rolipram or Scopolamine or Selegiline or Sertraline or Setiptiline or Teciptiline or Thozalinone or Tianeptin* or Toloxatone or Tranylcypromin* or Trazodone or Trimipramin* or Tryptophan* or Venlafaxine or Viloxazine or Vilazodone or Viqualine or Zalospirone).tw.  14. or/1-13  15. exp Craniomandibular Disorders/  16. exp Myofascial Pain Syndromes/  17. ((masticat* or myofasc* or orofacial*) and (pain* or dysfunction* or syndrom*)).mp.  18. (temporomandibular* or temporo-mandibular* or craniomandibular* or cranio-mandibular*).mp.  19. (facial pain adj3 (psychogenic* or atypical or chronic)).mp.  20. (tmj* or cmd* or tmd* or facial arthromyalgia*).mp.  21. (mpds not (myeloprolif* or myelo-prolif*)).mp.  22. or/15-21  23. ((randomized controlled trial or controlled clinical trial).pt. or (Randomized or placebo or randomly or trial or groups).ab. or drug therapy.fs.) not (exp animals/ not exp humans/)  24. 14 and 22 and 23  25. limit 24 to english language |
| Embase  Ovid Embase 1974 to 2024 April 19 | 1. exp antidepressant agent/  2. exp monoamine oxidase inhibitor/  3. exp neurotransmitter uptake inhibitor/  4. ((serotonin or norepinephrine or noradrenaline or neurotransmitter* or dopamin*) and (uptake or reuptake or re uptake)).tw.  5. (noradrenerg* or antiadrenergic or anti adrenergic or SSRI* or SNRI* or NARI* or SARI* or NDRI* or TCA* or tricyclic* or tetracyclic* or heterocyclic or pharmacotherap* or psychotropic).tw.  6. (antidepress* or anti-depress*).tw.  7. (MAOI* or RIMA).tw.  8. monoamine oxidase inhibit*.tw.  9. (Agomelatine or Amoxapine or Amineptine or Amitriptylin* or Amitriptylinoxide or Atomoxetine or Befloxatone or Benactyzine or Brofaromin*).tw.  10. (Bupropion or Amfebutamone or Butriptylin* or Caroxazone or Cianopramin* or Cilobamin* or Cimoxatone or Citalopram or Chlorimipramin* or Clomipramin* or Chlomipramin* or Clomipramine).tw.  11. (Clorgyline or Clovoxamin* or "CX157" or Tyrima or Tririma or Demexiptilin* or Deprenyl or Desipramin* or Pertofrane or Desvenlafaxine or Dibenzepin or Diclofensin* or Dimetacrin* or Dosulepin or Dothiepin or Doxepin or Duloxetine or Desvenlafaxine or "DVS-233" or Escitalopram or Etoperidone or Femoxetin* or Fluotracen or Fluoxetine or Fluvoxamin*).tw.  12. (Hyperforin or Hypericum or St John* or Imipramin* or Iprindole or Iproniazid* or Ipsapirone or Isocarboxazid* or Levomilnacipran or Lofepramin* or "Lu AA21004" or Vortioxetine or "Lu AA24530" or Tedatioxetine or "LY2216684" or Edivoxetine or Maprotilin* or Medifoxamin* or Melitracen or Metapramin* or Mianserin or Milnacipran or Minaprin* or Mirtazapin* or Moclobemide).tw.  13. (Nefazodone or Nialamide or Nitroxazepine or Nomifensin* or Norfenfluramin* or Nortriptylin* or Noxiptilin* or Opipramol or Paroxetine or Phenelzine or Pheniprazine or Pipofezine or Pirlindole or Pivagabine or Pizotyline or Propizepine or Protriptylin* or Quinupramine or Reboxetine or Rolipram or Scopolamine or Selegiline or Sertraline or Setiptiline or Teciptiline or Thozalinone or Tianeptin* or Toloxatone or Tranylcypromin* or Trazodone or Trimipramin* or Tryptophan* or Venlafaxine or Viloxazine or Vilazodone or Viqualine or Zalospirone).tw.  14. or/1-13  15. exp temporomandibular joint disorder/  16. exp myofascial pain/  17. ((masticat* or myofasc* or orofacial*) and (pain* or dysfunction* or syndrom*)).mp.  18. (temporomandibular* or temporo-mandibular* or craniomandibular* or cranio-mandibular*).mp.  19. (facial pain adj3 (psychogenic* or atypical or chronic)).mp.  20. (tmj* or cmd* or tmd* or facial arthromyalgia*).mp.  21. (mpds not (myeloprolif* or myelo-prolif*)).mp.  22. or/15-21  23. (Randomized controlled trial/ or Controlled clinical study/ or random*.ti,ab. or randomization/ or intermethod comparison/ or placebo.ti,ab. or (compare or compared or comparison).ti. or ((evaluated or evaluate or evaluating or assessed or assess) and (compare or compared or comparing or comparison)).ab. or (open adj label).ti,ab. or ((double or single or doubly or singly) adj (blind or blinded or blindly)).ti,ab. or double blind procedure/ or parallel group*1.ti,ab. or (crossover or cross over).ti,ab. or ((assign* or match or matched or allocation) adj5 (alternate or group*1 or intervention*1 or patient*1 or subject*1 or participant*1)).ti,ab. or (assigned or allocated).ti,ab. or (controlled adj7 (study or design or trial)).ti,ab. or (volunteer or volunteers).ti,ab. or human experiment/ or trial.ti.) not (((random* adj sampl* adj7 ("cross section*" or questionnaire*1 or survey* or database*1)).ti,ab. not (comparative study/ or controlled study/ or randomi?ed controlled.ti,ab. or randomly assigned.ti,ab.)) or (Cross-sectional study/ not (randomized controlled trial/ or controlled clinical study/ or controlled study/ or randomi?ed controlled.ti,ab. or control group*1.ti,ab.)) or (((case adj control*) and random*) not randomi?ed controlled).ti,ab. or (Systematic review not (trial or study)).ti. or (nonrandom* not random*).ti,ab. or "Random field*".ti,ab. or (random cluster adj3 sampl*).ti,ab. or ((review.ab. and review.pt.) not trial.ti.) or ("we searched".ab. and (review.ti. or review.pt.)) or "update review".ab. or (databases adj4 searched).ab. or ((rat or rats or mouse or mice or swine or porcine or murine or sheep or lambs or pigs or piglets or rabbit or rabbits or cat or cats or dog or dogs or cattle or bovine or monkey or monkeys or trout or marmoset*1).ti. and animal experiment/) or (Animal experiment/ not (human experiment/ or human/)))  24. 14 and 22 and 23  25. limit 24 to english language |
| Cochrane Library  via Wiley  Trials | #1 [mh "ANTIDEPRESSIVE AGENTS"]  #2 [mh "MONOAMINE OXIDASE INHIBITORS"]  #3 [mh "NEUROTRANSMITTER UPTAKE INHIBITORS"]  #4 ((serotonin or norepinephrine or noradrenaline or neurotransmitter* or dopamin*) and (uptake or reuptake or re uptake)):ti,ab  #5 (noradrenerg* or antiadrenergic or "anti adrenergic" or SSRI* or SNRI* or NARI* or SARI* or NDRI* or TCA* or tricyclic* or tetracyclic* or heterocyclic or pharmacotherap* or psychotropic):ti,ab  #6 antidepress* or anti-depress*  #7 (MAOI* or RIMA):ti,ab  #8 monoamine oxidase NEXT inhibit*  #9 (Agomelatine or Amoxapine or Amineptine or Amitriptylin* or Amitriptylinoxide or Atomoxetine or Befloxatone or Benactyzine or Brofaromin*):ti,ab  #10 (Bupropion or Amfebutamone or Butriptylin* or Caroxazone or Cianopramin* or Cilobamin* or Cimoxatone or Citalopram or Chlorimipramin* or Clomipramin* or Chlomipramin* or Clomipramine):ti,ab  #11 (Clorgyline or Clovoxamin* or "CX157" or Tyrima or Tririma or Demexiptilin* or Deprenyl or Desipramin* or Pertofrane or Desvenlafaxine or Dibenzepin or Diclofensin* or Dimetacrin* or Dosulepin or Dothiepin or Doxepin or Duloxetine or Desvenlafaxine or "DVS-233" or Escitalopram or Etoperidone or Femoxetin* or Fluotracen or Fluoxetine or Fluvoxamin*):ti,ab  #12 (Hyperforin or Hypericum or (St NEXT John*) or Imipramin* or Iprindole or Iproniazid* or Ipsapirone or Isocarboxazid* or Levomilnacipran or Lofepramin* or "Lu AA21004" or Vortioxetine or "Lu AA24530" or Tedatioxetine or "LY2216684" or Edivoxetine or Maprotilin* or Medifoxamin* or Melitracen or Metapramin* or Mianserin or Milnacipran or Minaprin* or Mirtazapin* or Moclobemide):ti,ab  #13 (Nefazodone or Nialamide or Nitroxazepine or Nomifensin* or Norfenfluramin* or Nortriptylin* or Noxiptilin* or Opipramol or Paroxetine or Phenelzine or Pheniprazine or Pipofezine or Pirlindole or Pivagabine or Pizotyline or Propizepine or Protriptylin* or Quinupramine or Reboxetine or Rolipram or Scopolamine or Selegiline or Sertraline or Setiptiline or Teciptiline or Thozalinone or Tianeptin* or Toloxatone or Tranylcypromin* or Trazodone or Trimipramin* or Tryptophan* or Venlafaxine or Viloxazine or Vilazodone or Viqualine or Zalospirone):ti,ab  #14 {OR #1-#13}  #15 [mh "Craniomandibular Disorders"]  #16 [mh "Myofascial Pain Syndromes"]  #17 (masticat* or myofasc* or orofacial*) and (pain* or dysfunction* or syndrom*)  #18 temporomandibular* or temporo-mandibular* or craniomandibular* or cranio-mandibular*  #19 (facial pain NEAR/3 (psychogenic* or atypical or chronic))  #20 tmj* or cmd* or tmd* or (facial NEXT arthromyalgia*)  #21 (mpds NOT (myeloprolif* or myelo-prolif*))  #22 {OR #15-#21}  #23 #14 AND #22 |
| CINAHL | S1 (MH "Antidepressive Agents+")  S2 (MH "Monoamine Oxidase Inhibitors+")  S3 (MH "Neurotransmitter Uptake Inhibitors+")  S4 TI ( ((serotonin or norepinephrine or noradrenaline or neurotransmitter* or dopamin*) and (uptake or reuptake or "re uptake")) ) OR AB ( ((serotonin or norepinephrine or noradrenaline or neurotransmitter* or dopamin*) and (uptake or reuptake or "re uptake")) )  S5 (noradrenerg* or antiadrenergic or "anti adrenergic" or SSRI* or SNRI* or NARI* or SARI* or NDRI* or TCA* or tricyclic* or tetracyclic* or heterocyclic or pharmacotherap* or psychotropic)  S6 TI ( antidepress* or anti-depress* ) OR AB ( antidepress* or anti-depress* )  S7 TI ( MAOI* or RIMA ) OR AB ( MAOI* or RIMA )  S8 "monoamine oxidase inhibit*"  S9 Agomelatine or Amoxapine or Amineptine or Amitriptylin* or Amitriptylinoxide or Atomoxetine or Befloxatone or Benactyzine or Brofaromin*  S10 Bupropion or Amfebutamone or Butriptylin* or Caroxazone or Cianopramin* or Cilobamin* or Cimoxatone or Citalopram or Chlorimipramin* or Clomipramin* or Chlomipramin* or Clomipramine  S11 lorgyline or Clovoxamin* or "CX157" or Tyrima or Tririma or Demexiptilin* or Deprenyl or Desipramin* or Pertofrane or Desvenlafaxine or Dibenzepin or Diclofensin* or Dimetacrin* or Dosulepin or Dothiepin or Doxepin or Duloxetine or Desvenlafaxine or "DVS-233" or Escitalopram or Etoperidone or Femoxetin* or Fluotracen or Fluoxetine or Fluvoxamin*  S12 TI ( Hyperforin or Hypericum or "St John*" or Imipramin* or Iprindole or Iproniazid* or Ipsapirone or Isocarboxazid* or Levomilnacipran or Lofepramin* or "Lu AA21004" or Vortioxetine or "Lu AA24530" or Tedatioxetine or "LY2216684" or Edivoxetine or Maprotilin* or Medifoxamin* or Melitracen or Metapramin* or Mianserin or Milnacipran or Minaprin* or Mirtazapin* or Moclobemide ) OR AB ( Hyperforin or Hypericum or "St John*" or Imipramin* or Iprindole or Iproniazid* or Ipsapirone or Isocarboxazid* or Levomilnacipran or Lofepramin* or "Lu AA21004" or Vortioxetine or "Lu AA24530" or Tedatioxetine or "LY2216684" or Edivoxetine or Maprotilin* or Medifoxamin* or Melitracen or Metapramin* or Mianserin or Milnacipran or Minaprin* or Mirtazapin* or Moclobemide ) Show Less  S13 (Nefazodone or Nialamide or Nitroxazepine or Nomifensin* or Norfenfluramin* or Nortriptylin* or Noxiptilin* or Opipramol or Paroxetine or Phenelzine or Pheniprazine or Pipofezine or Pirlindole or Pivagabine or Pizotyline or Propizepine or Protriptylin* or Quinupramine or Reboxetine or Rolipram or Scopolamine or Selegiline or Sertraline or Setiptiline or Teciptiline or Thozalinone or Tianeptin* or Toloxatone or Tranylcypromin* or Trazodone or Trimipramin* or Tryptophan* or Venlafaxine or Viloxazine or Vilazodone or Viqualine or Zalospirone)  S14 S1 OR S2 OR S3 OR S4 OR S5 OR S6 OR S7 OR S8 OR S9 OR S10 OR S11 OR S12 OR S13  S15 (MH "Craniomandibular Disorders+")  S16 (MH "Myofascial Pain Syndromes+")  S17 ((masticat* or myofasc* or orofacial*) and (pain* or dysfunction* or syndrom*))  S18 (temporomandibular* or temporo-mandibular* or craniomandibular* or cranio-mandibular*)  S19 ("facial pain" N3 (psychogenic* or atypical or chronic))  S20 tmj* or cmd* or tmd* or "facial arthromyalgia"  S21 (mpds NOT (myeloprolif* or myelo-prolif*))  S22 S15 OR S16 OR S17 OR S18 OR S19 OR S20 OR S21  S23 ( MH ( randomized controlled trials OR double‐blind studies OR single‐blind studies OR random assignment OR pretest‐posttest design OR cluster sample ) OR TI ( randomised OR randomized ) OR AB random* OR TI trial OR ( (MH (sample size) AND AB (assigned OR allocated OR control)) ) OR MH ( placebos OR crossover design OR comparative studies ) OR AB ( (control W5 group) OR (cluster W3 RCT) OR PT (randomized controlled trial)) ) NOT ( ( MH animals+ OR MH (animal studies) OR TI (animal model*) ) NOT MH (human) )  S24 S14 AND S22 AND S23  Limiter: English language |
| Scopus | TITLE-ABS-KEY ( ( ( serotonin OR norepinephrine OR noradrenaline OR neurotransmitter* OR dopamin* ) AND ( uptake OR reuptake OR "re uptake" ) ) OR noradrenerg* OR antiadrenergic OR "anti adrenergic" OR ssri* OR snri* OR nari* OR sari* OR ndri* OR tca* OR tricyclic* OR tetracyclic* OR heterocyclic OR pharmacotherap* OR psychotropic OR antidepress* OR anti-depress* OR maoi* OR rima OR "monoamine oxidase inhibit*" OR agomelatine OR amoxapine OR amineptine OR amitriptylin* OR amitriptylinoxide OR atomoxetine OR befloxatone OR benactyzine OR brofaromin* OR bupropion OR amfebutamone OR butriptylin* OR caroxazone OR cianopramin* OR cilobamin* OR cimoxatone OR citalopram OR chlorimipramin* OR clomipramin* OR chlomipramin* OR clomipramine OR clorgyline OR clovoxamin* OR "CX157" OR tyrima OR tririma OR demexiptilin* OR deprenyl OR desipramin* OR pertofrane OR desvenlafaxine OR dibenzepin OR diclofensin* OR dimetacrin* OR dosulepin OR dothiepin OR doxepin OR duloxetine OR desvenlafaxine OR "DVS-233" OR escitalopram OR etoperidone OR femoxetin* OR fluotracen OR fluoxetine OR fluvoxamin* OR hyperforin OR hypericum OR "St John*" OR imipramin* OR iprindole OR iproniazid* OR ipsapirone OR isocarboxazid* OR levomilnacipran OR lofepramin* OR "Lu AA21004" OR vortioxetine OR "Lu AA24530" OR tedatioxetine OR "LY2216684" OR edivoxetine OR maprotilin* OR medifoxamin* OR melitracen OR metapramin* OR mianserin OR milnacipran OR minaprin* OR mirtazapin* OR moclobemide OR nefazodone OR nialamide OR nitroxazepine OR nomifensin* OR norfenfluramin* OR nortriptylin* OR noxiptilin* OR opipramol OR paroxetine OR phenelzine OR pheniprazine OR pipofezine OR pirlindole OR pivagabine OR pizotyline OR propizepine OR protriptylin* OR quinupramine OR reboxetine OR rolipram OR scopolamine OR selegiline OR sertraline OR setiptiline OR teciptiline OR thozalinone OR tianeptin* OR toloxatone OR tranylcypromin* OR trazodone OR trimipramin* OR tryptophan* OR venlafaxine OR viloxazine OR vilazodone OR viqualine OR zalospirone ) AND ( TITLE-ABS-KEY ( ( masticat* OR myofasc* OR orofacial* ) AND ( pain* OR dysfunction* OR syndrom* ) ) OR TITLE-ABS-KEY ( temporomandibular* OR temporo-mandibular* OR craniomandibular* OR cranio-mandibular* OR tmj* OR cmd* OR tmd* OR "facial arthromyalgia*" ) OR TITLE-ABS-KEY ( "facial pain" W/3 ( psychogenic* OR atypical OR chronic ) ) OR TITLE-ABS-KEY ( mpds AND NOT ( myeloprolif* OR myelo-prolif* ) ) ) AND TITLE-ABS-KEY ( {Clinical-trial} OR {controlled-trial} OR randomi* OR randomly OR ( random W/4 ( allocat* OR distribut* OR assign* ) ) OR {placebo} OR {trial} OR {groups} OR {subgroups} ) OR TITLE ( rct ) AND ( LIMIT-TO ( LANGUAGE , "English" ) ) |
| Web of Science Core Collection | TS= ( ( ( serotonin OR norepinephrine OR noradrenaline OR neurotransmitter* OR dopamin* ) AND ( uptake OR reuptake OR "re uptake" ) ) OR noradrenerg* OR antiadrenergic OR "anti adrenergic" OR ssri* OR snri* OR nari* OR sari* OR ndri* OR tca* OR tricyclic* OR tetracyclic* OR heterocyclic OR pharmacotherap* OR psychotropic OR antidepress* OR anti-depress* OR maoi* OR rima OR "monoamine oxidase inhibit*" OR agomelatine OR amoxapine OR amineptine OR amitriptylin* OR amitriptylinoxide OR atomoxetine OR befloxatone OR benactyzine OR brofaromin* OR bupropion OR amfebutamone OR butriptylin* OR caroxazone OR cianopramin* OR cilobamin* OR cimoxatone OR citalopram OR chlorimipramin* OR clomipramin* OR chlomipramin* OR clomipramine OR clorgyline OR clovoxamin* OR "CX157" OR tyrima OR tririma OR demexiptilin* OR deprenyl OR desipramin* OR pertofrane OR desvenlafaxine OR dibenzepin OR diclofensin* OR dimetacrin* OR dosulepin OR dothiepin OR doxepin OR duloxetine OR desvenlafaxine OR "DVS-233" OR escitalopram OR etoperidone OR femoxetin* OR fluotracen OR fluoxetine OR fluvoxamin* OR hyperforin OR hypericum OR "St John*" OR imipramin* OR iprindole OR iproniazid* OR ipsapirone OR isocarboxazid* OR levomilnacipran OR lofepramin* OR "Lu AA21004" OR vortioxetine OR "Lu AA24530" OR tedatioxetine OR "LY2216684" OR edivoxetine OR maprotilin* OR medifoxamin* OR melitracen OR metapramin* OR mianserin OR milnacipran OR minaprin* OR mirtazapin* OR moclobemide OR nefazodone OR nialamide OR nitroxazepine OR nomifensin* OR norfenfluramin* OR nortriptylin* OR noxiptilin* OR opipramol OR paroxetine OR phenelzine OR pheniprazine OR pipofezine OR pirlindole OR pivagabine OR pizotyline OR propizepine OR protriptylin* OR quinupramine OR reboxetine OR rolipram OR scopolamine OR selegiline OR sertraline OR setiptiline OR teciptiline OR thozalinone OR tianeptin* OR toloxatone OR tranylcypromin* OR trazodone OR trimipramin* OR tryptophan* OR venlafaxine OR viloxazine OR vilazodone OR viqualine OR zalospirone ) AND ( TS= ( ( masticat* OR myofasc* OR orofacial* ) AND ( pain* OR dysfunction* OR syndrom* ) ) OR TS= ( temporomandibular* OR temporo-mandibular* OR craniomandibular* OR cranio-mandibular* OR tmj* OR cmd* OR tmd* OR "facial arthromyalgia*" ) OR TS= ( "facial pain" NEAR/3 ( psychogenic* OR atypical OR chronic ) ) OR TS= ( mpds NOT ( myeloprolif* OR myelo-prolif* ) ) )  AND  TS=(randomised OR randomized OR randomisation OR randomization OR placebo* OR (random* AND (allocat* OR assign*) ) OR (blind* AND (single OR double OR treble OR triple) )) NOT TS=(animal or animals or pisces or fish or fishes or catfish or catfishes or sheatfish or silurus or arius or heteropneustes or clarias or gariepinus or fathead minnow or fathead minnows or pimephales or promelas or cichlidae or trout or trouts or char or chars or salvelinus or salmo or oncorhynchus or guppy or guppies or millionfish or poecilia or goldfish or goldfishes or carassius or auratus or mullet or mullets or mugil or curema or shark or sharks or cod or cods or gadus or morhua or carp or carps or cyprinus or carpio or killifish or eel or eels or anguilla or zander or sander or lucioperca or stizostedion or turbot or turbots or psetta or flatfish or flatfishes or plaice or pleuronectes or platessa or tilapia or tilapias or oreochromis or sarotherodon or common sole or dover sole or solea or zebrafish or zebrafishes or danio or rerio or seabass or dicentrarchus or labrax or morone or lamprey or lampreys or petromyzon or pumpkinseed or pumpkinseeds or lepomis or gibbosus or herring or clupea or harengus or amphibia or amphibian or amphibians or anura or salientia or frog or frogs or rana or toad or toads or bufo or xenopus or laevis or bombina or epidalea or calamita or salamander or salamanders or newt or newts or triturus or reptilia or reptile or reptiles or bearded dragon or pogona or vitticeps or iguana or iguanas or lizard or lizards or anguis fragilis or turtle or turtles or snakes or snake or aves or bird or birds or quail or quails or coturnix or bobwhite or colinus or virginianus or poultry or poultries or fowl or fowls or chicken or chickens or gallus or zebra finch or taeniopygia or guttata or canary or canaries or serinus or canaria or parakeet or parakeets or grasskeet or parrot or parrots or psittacine or psittacines or shelduck or tadorna or goose or geese or branta or leucopsis or woodlark or lullula or flycatcher or ficedula or hypoleuca or dove or doves or geopelia or cuneata or duck or ducks or greylag or graylag or anser or harrier or circus pygargus or red knot or great knot or calidris or canutus or godwit or limosa or lapponica or meleagris or gallopavo or jackdaw or corvus or monedula or ruff or philomachus or pugnax or lapwing or peewit or plover or vanellus or swan or cygnus or columbianus or bewickii or gull or chroicocephalus or ridibundus or albifrons or great tit or parus or aythya or fuligula or streptopelia or risoria or spoonbill or platalea or leucorodia or blackbird or turdus or merula or blue tit or cyanistes or pigeon or pigeons or columba or pintail or anas or starling or sturnus or owl or athene noctua or pochard or ferina or cockatiel or nymphicus or hollandicus or skylark or alauda or tern or sterna or teal or crecca or oystercatcher or haematopus or ostralegus or shrew or shrews or sorex or araneus or crocidura or russula or european mole or talpa or chiroptera or bat or bats or eptesicus or serotinus or myotis or dasycneme or daubentonii or pipistrelle or pipistrellus or cat or cats or felis or catus or feline or dog or dogs or canis or canine or canines or otter or otters or lutra or badger or badgers or meles or fitchew or fitch or foumart or foulmart or ferrets or ferret or polecat or polecats or mustela or putorius or weasel or weasels or fox or foxes or vulpes or common seal or phoca or vitulina or grey seal or halichoerus or horse or horses or equus or equine or equidae or donkey or donkeys or mule or mules or pig or pigs or swine or swines or hog or hogs or boar or boars or porcine or piglet or piglets or sus or scrofa or llama or llamas or lama or glama or deer or deers or cervus or elaphus or cow or cows or bos taurus or bos indicus or bovine or bull or bulls or cattle or bison or bisons or sheep or sheeps or ovis aries or ovine or lamb or lambs or mouflon or mouflons or goat or goats or capra or caprine or chamois or rupicapra or leporidae or lagomorpha or lagomorph or rabbit or rabbits or oryctolagus or cuniculus or laprine or hares or lepus or rodentia or rodent or rodents or murinae or mouse or mice or mus or musculus or murine or woodmouse or apodemus or rat or rats or rattus or norvegicus or guinea pig or guinea pigs or cavia or porcellus or hamster or hamsters or mesocricetus or cricetulus or cricetus or gerbil or gerbils or jird or jirds or meriones or unguiculatus or jerboa or jerboas or jaculus or chinchilla or chinchillas or beaver or beavers or castor fiber or castor canadensis or sciuridae or squirrel or squirrels or sciurus or chipmunk or chipmunks or marmot or marmots or marmota or suslik or susliks or spermophilus or cynomys or cottonrat or cottonrats or sigmodon or vole or voles or microtus or myodes or glareolus or primate or primates or prosimian or prosimians or lemur or lemurs or lemuridae or loris or bush baby or bush babies or bushbaby or bushbabies or galago or galagos or anthropoidea or anthropoids or simian or simians or monkey or monkeys or marmoset or marmosets or callithrix or cebuella or tamarin or tamarins or saguinus or leontopithecus or squirrel monkey or squirrel monkeys or saimiri or night monkey or night monkeys or owl monkey or owl monkeys or douroucoulis or aotus or spider monkey or spider monkeys or ateles or baboon or baboons or papio or rhesus monkey or macaque or macaca or mulatta or cynomolgus or fascicularis or green monkey or green monkeys or chlorocebus or vervet or vervets or pygerythrus or hominoidea or ape or apes or hylobatidae or gibbon or gibbons or siamang or siamangs or nomascus or symphalangus or hominidae or orangutan or orangutans or pongo or chimpanzee or chimpanzees or pan troglodytes or bonobo or bonobos or pan paniscus or gorilla or gorillas or troglodytes)  Languages: English |
| Google Scholar | (antidepressants OR "neurotransmitter uptake inhibitors" OR "monoamine oxidase inhibitors") AND (temporomandibular OR temporo-mandibular) AND ("randomized controlled trial" OR RCT) |

Table S2: Excluded Articles in full-text phase selection and reasons for exclusion.

| **Study** | **Reason For Exclusion** |
| --- | --- |
| Harrison et al., 1997; Bendtsen & Jensen, 2000; Agius et al., 2013;  Haviv et al., 2019, Ivkovic et al., 2008. | Wrong patient population |
| Sorrel et al., 2003; Gupta et al., 2015; Bruti et al., 2021 | Wrong intervention |
| Plesh et al., 2000, Reisner, 2005; Goyal et al., 2020 | Wrong study design |

Figure S1- Percentage of total studies included in the systematic review categorized by an overall RoB assessment as low risk, some concerns, or high risk. The RoB was evaluated across 5 domains using the Cochrane RoB 2 tool.

**
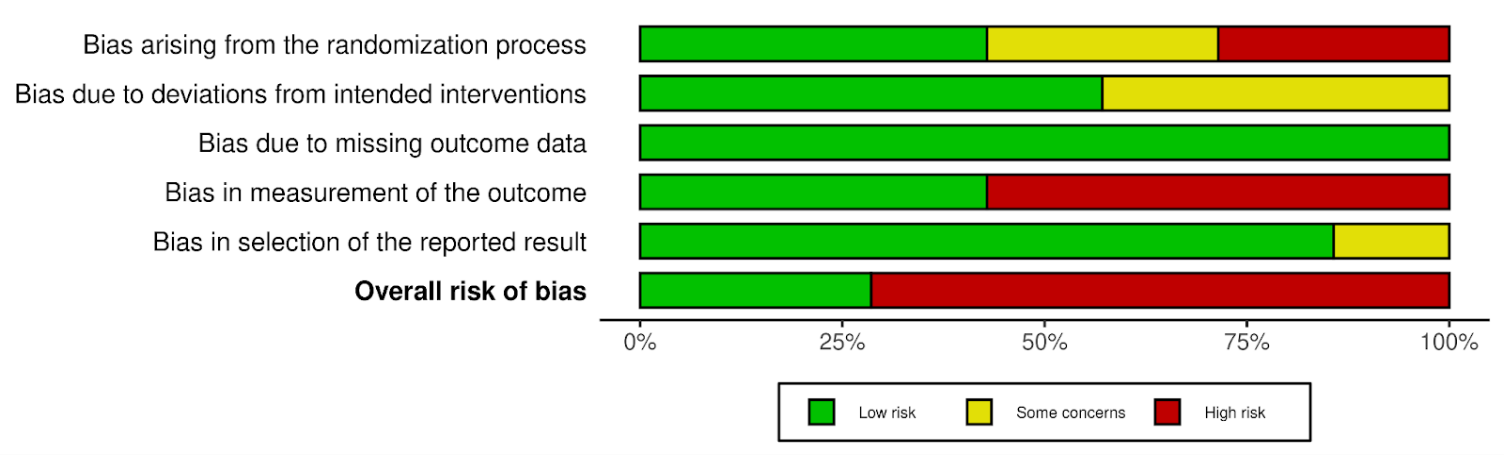
**
